# Supplementary material for: The interactome of intact mitochondria by cross-linking mass spectrometry provides evidence for coexisting respiratory supercomplexes
Source: Mol Cell Proteomics. 2017 Dec 8;17(2):216–32. doi: 10.1074/mcp.RA117.000470 (PMC5795388; doi:10.1074/mcp.RA117.000470)
Supplement: Supplemental Data [file supp_RA117.000470_133922_0_supp_23978_fzffwf.zip › spectra_annotation/mito_DR_spectra_annotation/295-1-4-1-7-1.pdf]

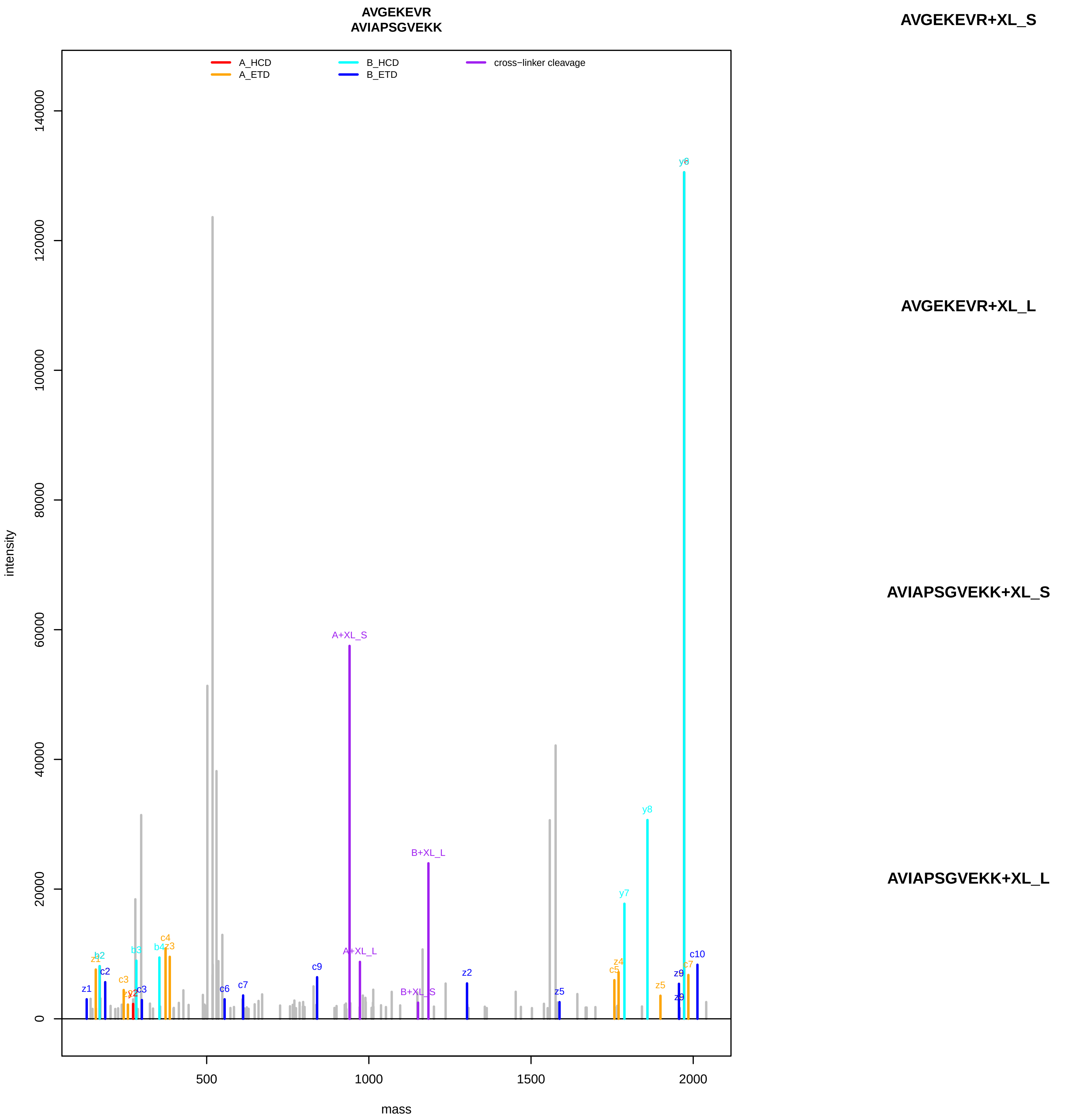

AVGEKEVR
AVIAPSGVEKK
A_HCD
A_ETD
B_HCD
B_ETD
cross−linker cleavage
intensity
mass
0
20000
40000
60000
80000
100000
120000
140000
500
1000
1500
2000
A+XL_S
A+XL_L
B+XL_L
B+XL_S
y8
y7
c10
c9
c7
c6
c4
c5
z2
z5
z4
z5
z9
b2
b3
b4
c2
c3
z1
z3
AVGEKEVR+XL_S
AVGEKEVR+XL_L
AVIAPSGVEKK+XL_S
AVIAPSGVEKK+XL_L
